# Assessment of intraventricular hemorrhage risk in preterm infants using mathematically simulated cerebral blood flow

**DOI:** 10.3389/fneur.2024.1465440

**Published:** 2024-10-18

**Authors:** Irina Sidorenko, Silke Brodkorb, Ursula Felderhoff-Müser, Esther Rieger-Fackeldey, Marcus Krüger, Nadia Feddahi, Andrey Kovtanyuk, Eva Lück, Renée Lampe

**Affiliations:** ^1^Department of Clinical Medicine, Center for Digital Health and Technology, Orthopedic Department, Research Unit for Pediatric Neuroorthopedics and Cerebral Palsy of the Buhl-Strohmaier Foundation, Klinikum rechts der Isar, School of Medicine and Health, Technical University of Munich, Munich, Germany; ^2^Clinic for Neonatology, Munich Clinic Harlaching & Schwabing, Munich, Germany; ^3^Department of Pediatrics I, Neonatology, Paediatric Intensive Care, Paediatric Infectious Diseases, Paediatric Neurology, Centre for Translational Neuro-and Behavioural Sciences, University Hospital Essen, Faculty of Medicine, University of Duisburg-Essen, Essen, Germany; ^4^Clinic and Policlinic for Neonatology, Klinikum rechts der Isar, School of Medicine and Health, Technical University of Munich, Munich, Germany; ^5^Markus Würth Professorship, Technical University of Munich, Munich, Germany

**Keywords:** preterm birth, immature brain, intraventricular hemorrhage, cerebral blood flow, multivariate logistic regression analysis, risk score, prognostic accuracy, ROC analysis

## Abstract

Intraventricular hemorrhage (IVH)4 is one of the most threatening neurological complications associated with preterm birth which can lead to long-term sequela such as cerebral palsy. Early recognition of IVH risk may prevent its occurrence and/or reduce its severity. Using multivariate logistic regression analysis, risk factors significantly associated with IVH were identified and integrated into risk scales. A special aspect of this study was the inclusion of mathematically calculated cerebral blood flow (CBF) as an independent predictive variable in the risk score. Statistical analysis was based on clinical data from 254 preterm infants with gestational age between 23 and 30 weeks of pregnancy. Several risk scores were developed for different clinical situations. Their efficacy was tested using ROC analysis, and validation of the best scores was performed on an independent cohort of 63 preterm infants with equivalent gestational age. The inclusion of routinely measured clinical parameters significantly improved IVH prediction compared to models that included only obstetric parameters and medical diagnoses. In addition, risk assessment with numerically calculated CBF demonstrated higher predictive power than risk assessments based on standard clinical parameters alone. The best performance in the validation cohort (with AUC = 0.85 and TPR = 0.94 for severe IVH, AUC = 0.79 and TPR = 0.75 for all IVH grades and FPR = 0.48 for cases without IVH) was demonstrated by the risk score based on the MAP, pH, CRP, CBF and leukocytes count.

## 1 Introduction

Advances in obstetric and neonatal care in recent decades have resulted in a significant reduction in perinatal mortality. However, the incidence of intraventricular hemorrhage (IVH) in preterm infants with gestational age (GA) less than 32 weeks of pregnancy or body weight at birth (BW) less than 1,500 g remains still very high. It reaches 40% (1) for preterm infants and increases for lower gestational age, reaching 52% for very preterm infants born before 28 weeks gestation (WG) (2). IVH is one of the most threatening complications of preterm birth, which may lead to death or long term severe neurological disorders, such as motor, sensor and/or cognitive disabilities. It is caused by rupture of the fragile vascular vessels within the germinal matrix, a highly vascularised layer located above the caudate nucleus and composed of glial and neuronal progenitor cells (3). The prediction of IVH and development of prevention strategies are among the most important tasks of modern neonatal care.

The underlying cause of cerebral hemorrhage in preterm infants is multifactorial. Risk factors can be divided into several groups. The first group includes pregnancy pathologies that are usually known before delivery. The second group consists of birth characteristics, including Apgar score, which are known already in the first minutes of life. The most recognized risk factors associated with IVH are low gestational age, low birth weight and low Apgar scores (2, 3). The third group contains medical diseases, among which the perinatal infection and early sepsis are the most dangerous (1, 2). This parameters can be included in risk models as soon as diseases have been diagnosed. The fourth group consists of medical parameters that are determined through routine laboratory tests, blood gas analysis and regular observations. These measurements are usually performed several times a day as part of the standard monitoring of preterm infants. Due to the regularity of the investigations, these parameters provide valuable information about the patient’s current status.

Another risk factor associated with the low gestational age is immaturity of the cerebral vascular system. The presence of germinal matrix before 32 WG plays a crucial role in the development of IVH (3). The weakness of the cytoskeletal structure exposes the delicate vasculature of the germinal matrix to an increased risk of rupture. Another anatomical feature of germinal matrix vessels is that capillaries are larger in diameter and the muscular layer of the vessels is poorly developed or absent. Thus, these anatomic features make the vessels susceptible to rupture and increase the risk of cerebral hemorrhage in early childhood. In addition, autoregulation of immature cerebral vessels is underdeveloped (4), resulting in an inadequate response to cerebral blood flow (CBF) fluctuations. Thus, critical CBF values are additional risk factors for IVH (3, 4), and taking them into account could potentially improve prognostic models.

Since gestational age, birth weight and Apgar score are often not sufficient to determine the health condition of preterm neonates, several multidimensional scales have been developed to assess risks for mortality or survival (5). The most popular scores, such as CRIB (clinical risk index for babies), CRIB-II (6, 7), SNAP (score for neonatal acute physiology), SNAP-II (8, 9), SNAPPE (score for neonatal acute physiology with perinatal extension) (10), PREM (prematurity risk evaluation measure) (11), were initially validated as predictors of mortality and morbidity, however they also have been shown to predict severe IVH more accurately than BW or GA alone (12). While low GA and BW alone are definitely important predictors for IVH, several other score systems for predicting early risk for severe hemorrhage based on various medical parameters have been developed (1, 13, 14).

All of the above mentioned methods have primarily included prenatal and perinatal variables, neonatal diseases and clinical parameters available within the first hours of life, arguing that the vast majority of IVH occurs within the first 48 h. Although numerous perinatal, obstetric and neonatal risk factors associated with IVH may be identified early after birth (15), recent studies have revealed a significant association between IVH and some routinely recorded clinical parameters such as systolic and diastolic arterial blood and respiratory data (16–18). Additionally, it was demonstrated that fluctuating CBF had a significant association with IVH (19, 20). Furthermore, CBF was an important variable in machine learning models for differentiation between preterm infants with and without IVH (18).

Despite existing non-invasive methods to measure CBF, such as xenon-133 (21), near-infrared spectroscopy (NIRS) (22), diffusion correlation spectroscopy (DCS) (23) and others, CBF is not yet a routinely measured parameter in monitoring preterm infants in the neonatal intensive care unit (NICU). The mathematical model for calculating CBF from standard clinical records of mean arterial pressure (MAP), partial pressure of carbon dioxide (pCO_2_), partial pressure of oxygen (pO_2_), and hematocrit (Ht) were in good agreement with experimental measurements (19, 24) and can therefore be used to analyze IVH risk factors instead of measured values.

The main aim of this study was to develop predictive models for different clinical situations to identify preterm infants at increased risk of IVH using standard pre/postnatal and birth parameters, medical diagnoses and routinely measured parameters. We also investigated a possibility to enhance a prognostic accuracy of the IVH risk scores by including numerically calculated CBF as independent predictive variable.

## 2 Materials and methods

### 2.1 Study population and data collection

The present work is a retrospective study based on clinical data of two cohorts of preterm infants with gestational age 23–30 WG born in four German hospitals with the highest level of care according to the German regulations. The study was approved by the ethic committee of School of Medicine Klinikum rechts der Isar, Technical University of Munich (Ref. 364/15), and Ethic Committee of University Hospital Essen, University Duisburg-Essen (Ref. 16-7284-BO).

The derivation cohort used for construction of IVH risk scores included 254 preterm infants born between 2006 and 2016. The prediction accuracy of the developed risk scores was validated on more recent population of 63 preterm infants (validation cohort) born between 2017 and 2019.

In all four settings, occurrence of IVH was diagnosed by cranial ultrasound performed routinely on day 1, 3, 7, 14 of life and more frequently (up to daily) in case of suspected hemorrhage. The severity of IVH was divided into four grades according to Papile classification (25). In the study, groups of patients with and without IVH diagnosis are referred to as the affected and control groups.

In both cohorts, the following clinical data were collected:

- Pregnancy pathologies: (1) EPH-gestosis/pre-eclampsia, (2) preterm premature rupture of membranes (PPROM), (3) chorioamnionitis/intra-amniotic infection syndrome, (4) intrauterine growth restriction (IUGR), (5) *in vitro* fertilization (IVF), (6) feto-fetal transfusion syndrome (FFTS).
- Birth parameters: (1) delivery mode (spontaneous or by cesarean section), (2) sex, (3) birth weight, (4) gestational age, (5) singleton/multiple births, (6) Apgar at 1 min, (7) Apgar at 5 min, (8) Apgar at 10 min.
- Medical diagnoses: (1) respiratory distress syndrome (RDS), (2) sepsis, (3) pulmonary hemorrhage, (4) erythrocyte transfusion, (5) acidosis (metabolic and/or respiratory), (6) focal/spontaneous intestinal perforation (FIP/SIP), (7) thrombocytopenia, (8) necrotizing enterocolitis (NEC), (9) cholestasis, (10) cardiopulmonary adaptation disorder.
- Routinely measured parameters (this group of parameters consists of measurements provided by routine laboratory tests, blood gas analyses and regular observations, which are usually carried out several times a day as part of the standard monitoring of premature babies): (1) pH, (2) pCO_2_, (3) pO_2_, (4) MAP, (5) Ht, (6) leukocyte count, (7) thrombocyte count, (8) C-reactive protein (CRP).
- Additionally, within the framework of this study, the CBF and CBF-CBF_mean_ are calculated for each set of measurements of MAP, pCO_2_, pO_2_ and Ht and treated as independent parameters in the statistical analyses.

The six pregnancy pathologies and eight birth parameters were recorded shortly after birth, further 10 infant diagnoses were recorded during postnatal care. In addition, eight routinely measured clinical parameters were systematically recorded during 7 days before and 3 days after IVH diagnose, or 10 days after birth for preterm infants without IVH diagnosis.

### 2.2 Calculation of the cerebral blood flow

In the present study we calculate CBF from the 6 medical parameters, namely WG, BW, MAP, pCO_2_, pO_2_ and Ht using the hierarchical cerebral vascular model that was initially proposed for adult brain (26) and then adjusted to the peculiarities of the immature brain (19, 27). In the model, the cerebral vascular system is represented by 19 serially connected levels, each containing parallel connected vessels of a certain size. At each level, the number of vessels and their length and diameter are scaled according to the patient’s gestational age and the brain weight estimated from the birth weight (28). At the capillary level, an additional area is added as a parallel circuit with the number of vessels corresponding to the relative volume of the germinal matrix at a given gestational age (29). Furthermore, the effect of Ht on blood viscosity is included in the calculation of vascular resistance (30, 31) and autoregulation activity of the brain vessels (vasoconstriction and vasodilation) is accounted by increasing and decreasing of vessel’s diameter according to the measured values of MAP, pCO_2_ and pO_2_ (19, 24, 27).

The calculated CBF value was included in the statistical analyses as an independent parameter. In addition, the mean value of CBF for the group of infants without IVH was calculated for each WG. The obtained CBF_mean_ value was used as a reference value of the optimal CBF at each WG and the CBF-CBF_mean_ value was included as a further predictive variable in the statistical analyses.

### 2.3 Statistical methods

To build a risk score, multivariable stepwise logistic regression analysis was applied (1, 13). In our study, IVH diagnosis plays a role of response variable *y*, which has a binary type. The predictive variables (predictors) *x_j_* are medical characteristics, which have either continuous or binary type. The selection of potential variables for scoring system started with the analysis of parameters related to IVH. To detect IVH risk factors, a univariate analysis was performed using two-sided Wilcoxon’s rank-sum test for continuous and Fisher’s exact test for categorical parameters (32). A 5% significance level, which corresponds to *p*-value = 0.05, was taken as the threshold value for including each parameter as a candidate variable in the multivariate prediction model.

The parameters found to be associated with IVH were then ranked in respect to the response variable according to the *p*-values of chi-square test with null hypothesis that predictor and response variable are independent. The *p*-value <0.05 means that the predictor and response variable are significantly associated with each other. Predictors with larger values −log(*p*) were ranked higher and the value −log(0.05) = 3 was taken as the threshold for including them into the score model.

Incorporation of independent predictors into risk scores was performed using a generalized linear regression with logit link function as follows:


$$y=\frac{\exp\left(b_{0}+\sum_{j=1}^{N}\left(x_{j}b_{j}\right)\right)}{1+\exp\left(b_{0}+\sum_{j=1}^{N}\left(x_{j}b_{j}\right)\right)}$$

In order to prevent overfitting, we implemented stepwise predictors selection. We started with a single variable model (*N* = 1) using the highest ranked predictor and added lower ranking predictors after running the chi-squared test with the null hypothesis that deviance of old and new regression models are equal. A *p*-value <0.05 rejects the null hypothesis and means that new predictor significantly improves the fitting model and should be included in the score. Only predictors with statistically significant coefficients *b_j_* were included in the model, and only models that were statistically significantly different from the constant model were used further for construction of risk scores.

The coefficients *b_j_* obtained by the linear regression analyses were then integrated in a risk score as follows:


$$S\left(t\right)=b_{0}+\overset{N}{\underset{j=1}{\sum }}x_{j}\left(t\right)b_{j}$$

Here *x_j_ (t)* is the value of the predictor *j* averaged over all measurements up to time *t*. For early IVH prediction, as long as the measured clinical parameters are not available, only the parameters of groups I–III were considered in the regression model. For the prediction of IVH on the second day, the clinical parameters were averaged over the first day of life (measurements of preterms with IVH on the first day are then excluded), and for the later prediction of IVH, parameters were averaged over all days before IVH.

To evaluate the effectiveness of the risk scores constructed, the receiver operating characteristic (ROC) method for classification between the control and affected groups was applied (33, 34). For each patient from control and affected groups score value was calculated, then true positive rate (TPR) and false positive rate (FPR) (Table 1) were computed for different threshold values. The ROC curves were constructed by plotting TPR against FPR, and the area under the ROC curve (AUC) was estimated (35). The later provides a quantitative measure of predictive power of the score: perfect predictor has AUC = 1, which corresponds to TPR = 1 and FPR = 0, and predictor with AUC = 0.5 is equivalent to random choice. The threshold for optimal performance was chosen at the point on the ROC curve with the smallest distance to the upper left corner of the unit square (36) and used further for the normalization of the regression coefficient *b_0_*, so that the threshold for IVH risk was equal to 0 for all risk scores. The validation of the resulting IVH risk scores was performed by calculating the TPR and FPR using independent data from the validation cohort.

**Table 1:** Variables of ROC analyses.

Variable	Definition
P	The total number of patients with IVH
N	The total number of patients without IVH
TP	True positive (the number of correctly detected patients with IVH)
TN	True negative (the number of correctly detected patients without IVH)
FP	False positive (the number of patients without IVH detected as with IVH)
FN	False negative (the number of patients with IVH detected as without IVH)
TPR = TP/P	True positive rate
FPR = FP/N	False positive rate

Statistical analyses was carried out using standard library of MATLAB2024a. Observations with missing values were not used in analyses, medical parameters that were measured less often than others were extended until the next regular measurement. For calculation of CBF, missing parameter values were replaced with the latest measured value.

## 3 Results

### 3.1 Identification of risk parameters of IVH

Basic clinical characteristics of the derivation and validation cohorts are presented in Table 2. Here, continuous variables are expressed as mean and standard deviation, while binary variables are presented as the number of cases and percentages.

**Table 2:** Clinical characteristics of the study cohorts.

Clinical characteristic	Derivation cohort *n* = 254 (100%)			Validation cohort *n* = 63 (100%)		
	Min	Max	Mean ± Stdev	Min	Max	Mean ± Stdev
Gestational age [WG + days]	23	30 + 6	26.45 ± 2.11	23 + 1	30 + 6	26.39 ± 2.17
Birth weight [g]	335	1580	864.06 ± 279.1	490	1590	905.16 ± 279.65
Male sex	122 (48%)			39 (61.90%)		
Multiple birth	95 (37.4%)			25 (39.68%)		
Vaginal delivery	22 (8.66%)			7 (11.11%)		
IVH	136 (53.54%)			37 (58.73%)		
EPH-gestosis/pre-eclampsia	25 (9.84%)			2 (3.17%)		
PPROM	73 (28.74%)			32 (50.79%)		
Chorioamnionitis	117 (46.06%)			43 (68.25%)		
IUGR	14 (5.51%)			0		
IVF	32 (12.59%)			8 (12.69%)		
FFTS	8 (3.15%)			1 (1.59%)		
RDS	84 (33.07%)			14 (22.22%)		
Sepsis	120 (47.24%)			20 (31.75%)		
Pulmonary hemorrhage	21 (8.27%)			8 (12.69%)		
Erythrocyte transfusion	164 (64.57%)			39 (61.90%)		
Acidosis (metabolic and/or respiratory)	38 (14.96%)			10 (15.87%)		
FIP/SIP	23 (9.06%)			3 (4.76%)		
Thrombocytopenia	21 (8.27%)			4 (6.35%)		
NEC	20 (7.87%)			10 (15.87%)		
Cholestasis	12 (4.72%)			0		
Cardiopulmonary adaptation disorder	19 (7.48%)			18 (28.57%)		

To create a risk score, the data from the derivation cohort of 254 patients (136 with and 118 without diagnosis of IVH) was used. During data collection, the affected and control groups were matched according to gestational age and birth weight. Thus, these parameters had statistically equal (*p*-value >0.05) mean values (Table 3) and, therefore, were not used in this study as predictor variables for risk assessment.

**Table 3:** Comparison of gestational age and birth weight of affected and control groups.

Parameter	With IVH *n* = 136	No IVH *n* = 118	*p*-value
Gestational age	26.25 ± 2.05	26.68 ± 2.17	0.12
Birth weight	875.66 ± 300.5	850.68 ± 252.81	0.70

The statistical comparison of pregnancy pathologies, birth parameters and infant diagnoses in the affected and control groups of the derivation cohort is presented in Table 4. The table is sorted according to the *p*-value: the most significant parameters, i.e., those with the smallest *p*-value, are at the top. Seven parameters (EPH-gestosis/pre-eclampsia, PPROM, cardiopulmonary adaptation disorder, IUGR, IVF, RDS and FFTS) demonstrated inverse relationship with the development of IVH, i.e., the percentage of preterm infants with these diagnoses in the affected group was lower than in the control group. This can be explained by the effects of the medical treatment provided, which is out of scope of this study. Therefore, these parameters were not considered as predictor variables in the following risk assessment. Another seven parameters (sepsis, delivery mode, erythrocyte transfusion, chorioamnionitis, thrombocytopenia, NEC and multiple birth) had no significant association with IVH (i.e., *p*-value >0.5). Significant association with IVH (i.e., *p*-value <0.5) was revealed for the following 8 parameters: Apgar at 1 min, Apgar at 5 min, Apgar at 10 min, FIP/SIP, cholestasis, acidosis, male sex and pulmonary hemorrhage. The parameters found to be associated with IVH were further used as predictive variables in the logistic regression analyses.

**Table 4:** Pregnancy pathologies, birth parameters and infant diagnoses in the derivation cohort.

Parameter	With IVH *n* = 136 (100%)	No IVH *n* = 118 (100%)	*p*-value
**Apgar at 1 min**	**5.19 ± 2.25**	**6.21 ± 2.02**	**<0.001**
**Apgar at 5 min**	**6.80 ± 1.82**	**7.52 ± 1.41**	**<0.001**
**Apgar at 10 min**	**7.73 ± 1.37**	**8.37 ± 0.97**	**<0.001**
**FIP/SIP**	**20 (14.7%)**	**3 (2.54%)**	**<0.001**
**Cholestasis**	**11 (8.09%)**	**1 (0.85%)**	**0.01**
**Acidosis**	**27 (19.85%)**	**11 (9.32%)**	**0.02**
**Sex (male)**	**74 (54.4%)**	**48 (40.68%)**	**0.03**
**Pulmonary hemorrhage**	**16 (11.76%)**	**5 (4.24%)**	**0.04**
EPH-gestosis/pre-eclampsia	7 (5.18%)	18 (15.25%)	0.01
Sepsis	70 (51.47%)	50 (42.37%)	0.17
PPROM	34 (25.00%)	39 (33.05%)	0.17
Delivery mode (spontaneous birth)	15 (11.02%)	7 (5.93%)	0.18
Erythrocyte transfusion	93 (68.38%)	71 (60.17%)	0.19
Chorioamnionitis	67 (49.26%)	50 (42.37%)	0.31
Cardiopulmonary adaptation disorder	8 (5.88%)	11 (9.32%)	0.34
IUGR	6 (4.41%)	8 (6.78%)	0.43
IVF	15 (11.03%)	17 (14.41%)	0.45
Thrombocytopenia	13 (9.56%)	8 (6.78)	0.49
RDS	42 (30.88%)	42 (35.59%)	0.50
NEC	12 (8.82%)	8 (6.78%)	0.64
Multiple birth	52 (38.24%)	43 (36.44%)	0.79
FFTS	4 (2.94%)	4 (3.39)	1

Key information in the table is highlighted in bold.

### 3.2 Construction of IVH risk scores

**Figure 1:**
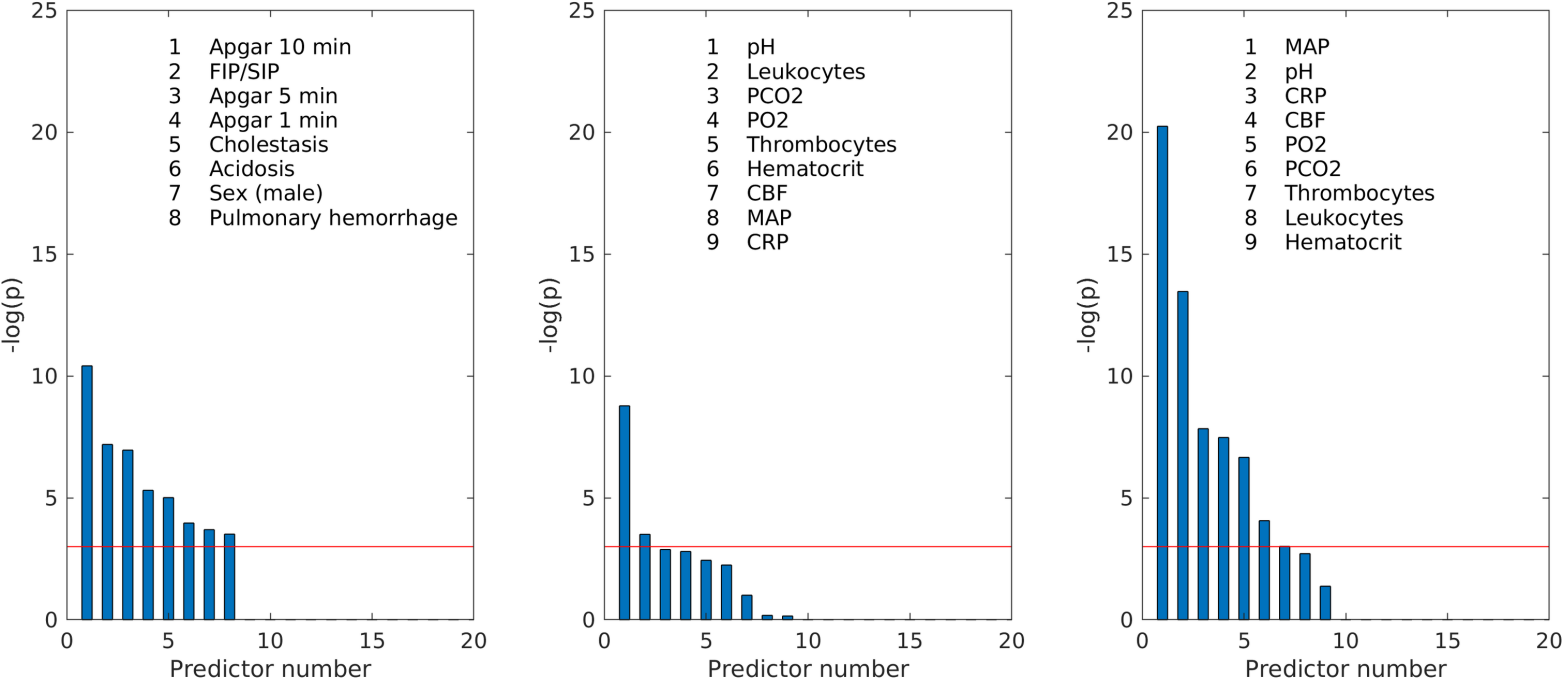
Ranking of the predictive variables according to their *p*-values of chi-square test: (left) medical parameters from Table 4 significantly associated with IVH; (middle) routinely measured parameters averaged over the first day of life; (right) routinely measured parameters averaged over all days before IVH in the affected groups and over 10 days of life in the control group. The red line corresponds to the significance threshold −log(*p*) = 3, which corresponds to the *p*-value = 0.05.

**Figure 2:**
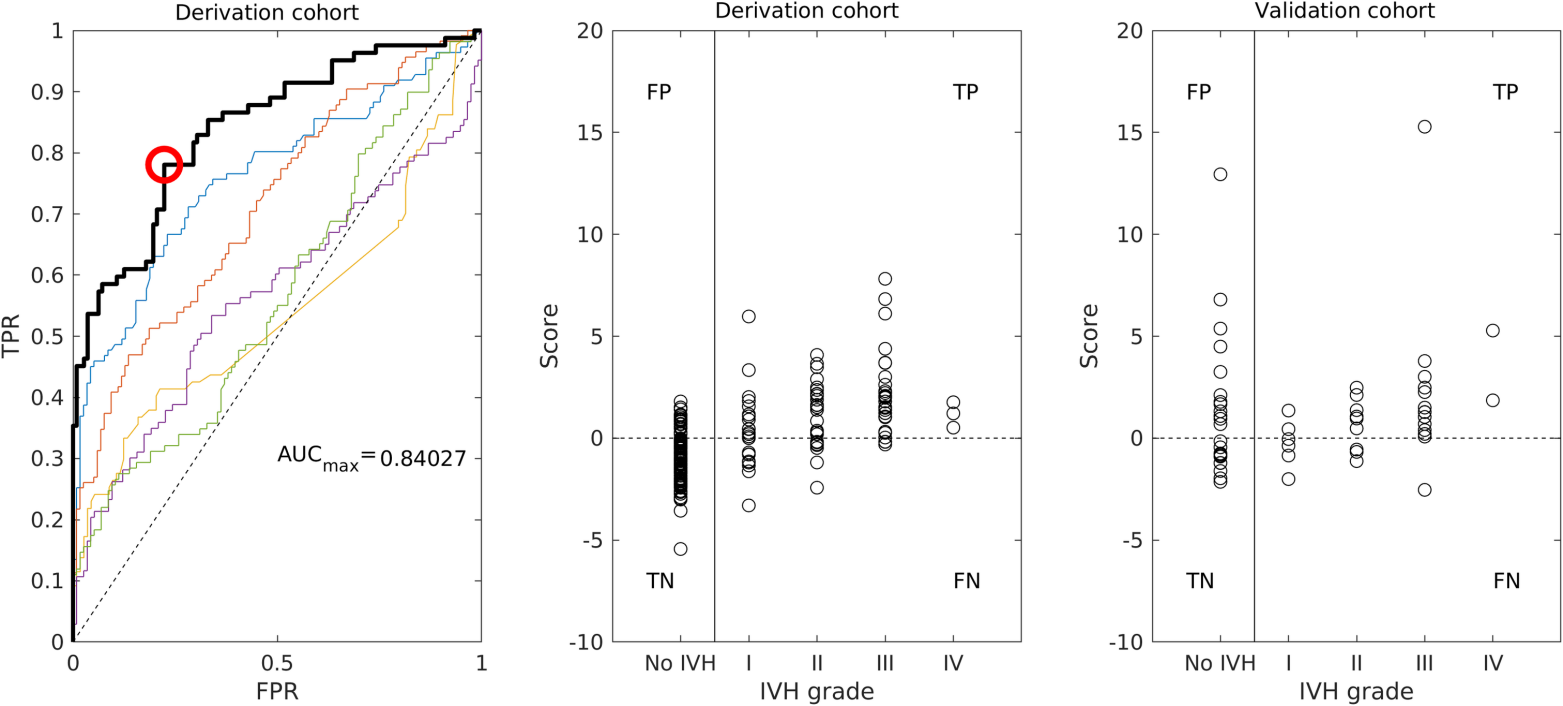
Performance of the IVH risk score S7 (see Tables 5, 6): ROC curve (left) and scatter plot (middle) for the derivation cohort; scatter plot for the validation cohort (right).

We started the construction of risk scores with the ranking of the predictive variables (Figure 1), which were then included stepwise in the multivariable regression analyses according to their significance. For different clinical situations, several scores were designed (Table 5). The performance of the resulting scores was evaluated using the ROC analysis. We determined the optimal threshold from the ROC curve for patients with all grades of IVH and then calculated the TPR and FPR for patients with all (I–IV) and severe (III–IV) grades of IVH (the example of ROC curve with optimal threshold and corresponding scatter plot is shown in Figure 2).

**Table 5:** Performance of the constructed IVH risk scores on the derivation cohort.

Score *b*_0 … 6_ *p*_value_0 … 6_	AUC		TPR		FPR
	IVH grades		IVH grades		
	I–IV	III–IV	I–IV	III–IV	
S1 = Apgar at 10 min + FIP/SIP + cholestasis + acidosis + male sex + pulmonary hemorrhage *b*_0 … 6_ = [1.8967; −0.3255; 1.8685; 2.0971; 0.7245; 0.7077; 1.0828] *p*-value_0 … 6_ = [0.0570; 0.0111; 0.0047; 0.0523; 0.0755; 0.0109; 0.0492]	0.74	0.78	0.55	0.64	0.17
S2 = Apgar at 10 min + pH + FIP/SIP + Leukocytes *b*_0 … 4_ = [102.4143; −0.3403; −13.5181; 2.0952; −0.0993] *p*-value_0 … 4_ = [<0.0001; 0.0360; <0.0001; 0.0234; <0.0001]	0.81	0.88	0.75	0.87	0.25
S3 = Apgar at 10 min + pH + Leukocytes *b*_0 … 3_ = [101.9569; −0.3345; −13.4425; −0.0970] *p*-value_0 … 3_ = [<0.0001; 0.0385; <0.0001; 0.0001]	0.79	0.86	0.78	0.90	0.29
S4 = MAP + pH + CRP + FIP/SIP + male sex + Leukocytes *b*_0 … 6_ = [108.5723; −0.1105; 14.3532; 0.4666; 2.2413; 0.7966; −0.0704] *p*-value_0 … 6_ = [0.0013; 0.0020; 0.0021; 0.0091; 0.0107; 0.0365; 0.0055]	0.84	0.94	0.77	0.91	0.19
S5 = MAP + pH + CRP + Leukocytes *b*_0 … 4_ = [117.5718; −0.0969; −15.5920; 0.5566; −0.06549] *p*-value_0 … 4_ = [0.0004; 0.0042; 0.0007; 0.0018; 0.0034]	0.82	0.93	0.72	0.88	0.21
S6 = MAP + pH + CRP + CBF + FIP/SIP + Leukocytes *b*_0 … 6_ = [85.6357; −0.1753; −10.9974; 0.4205; 0.1264; 2.0791; −0.0677] *p*-value_0 … 6_ = [0.0172; 0.0004; 0.0270; 0.0346; 0.0161; 0.0195; 0.0042]	0.85	0.94	0.79	0.91	0.23
S7 = MAP + pH + CRP + CBF + Leukocytes *b*_0 … 5_ = [85.9253; −0.1780; −11.0513; 0.4525; 0.1268; −0.0581] *p*-value_0 … 5_ = [0.0152; 0.0003, 0.0243; 0.0225; 0.0150; 0.0070]	0.84	0.94	0.77	0.91	0.22

For the initial prediction of IVH risk, we constructed the score S1 based only on pregnancy pathologies, birth parameters and infant diagnoses, which are usually known before measured parameters are available (Figure 1 left). Although eight parameters were significantly associated with IVH, two of them, namely Apgar at 1 min and Apgar at 5 min, were absent in the score due to their high correlation with Apgar at 10 min (Pearson correlation 0.61 and 0.77, respectively). The score S1 demonstrated only moderate performance (AUC = 0.74) and included several statistically insignificant coefficients, suggesting that this group of medical parameters was insufficient for effective IVH prediction.

The improvement of IVH prediction can be achieved by including routinely measured parameters in the regression model. In the first day of life, the availability of measurements is usually limited. To determine risk scores for first and second days of life, we averaged routinely measured parameters over the first day (patients with IVH on the first day are excluded from consideration at this stage). Thus, only pH and leukocyte count demonstrated significant association with IVH (Figure 1 middle). Two other parameters, CBF-CBF_min_ and pCO_2_, were close to the limit of significance. It is important to note, that mathematically calculated parameter CBF-CBF_min_ had stronger association with IVH than each of the parameters used for its calculation (Figure 1 middle). The integration of the significant measured parameters averaged over the first day of life (Figure 1 middle) in the regression procedure resulted in the risk models with 4 and 3 parameters (score S2 and S3 in Table 5), which both had only significant regression coefficients and much better performance in ROC analyses than S1 (Table 5).

To construct a risk score for prediction of IVH when the values of all measured parameters are already available, we averaged them for all days before IVH and added averaged values to the regression analysis according to their significance (Figure 1 right). In this case, the calculated CBF was ranked fourth among statistically significant parameters and had a stronger association with IVH than pCO_2_, pO_2_, and Ht, which are used for its calculation. Inclusion of the measured parameters averaged over all days before IVH improved the resulting risk scores (scores S4 and S5 in Table 5). Further improvement in risk score was achieved by adding the calculated CBF to the regression model (scores S6 and S7 in Table 5). The performance of the risk score S7 is illustrated on the Figure 2 (left: ROC curve with optimal threshold value; middle: scatter plot of score values of individual patient).

In order to demonstrate the advantage of including the calculated CBF in the risk model, we constructed a risk score without CBF but with all clinical parameters used in the mathematical model to calculate CBF. The performance of the resulting risk score (S = pH + Leukocytes + pO_2_ + pCO_2_ + MAD + Ht, not in Table 5) with AUC = 0.88 appeared to be worse than AUC = 0.94 of the risk score S7 which includes CBF. This result indicates that the mathematical model for CBF calculation provides complementary information that improves the prediction of IVH risk.

### 3.3 Validation of the constructed IVH risk scores

Clinical characteristics of the validation cohort of 63 preterm infants are presented in Table 2. For each patient in the validation cohort, score values with coefficients presented in Table 5 were calculated (Figure 2 right) and used to determine FPR for control group and TPR for all and severe grades of IVH (Table 6). The best performance was achieved by the scores, which included routinely measured parameters and calculated CBF (scores S5, S6 and S7). For this scores, correct identification of patients with IVH (TPR) in the validation cohort remained at the same level as in the derivation cohort. False identification of healthy patients to be at risk of IVH (FPR) in validation cohort was higher than in the derivation cohort. However, increase of FPR was smaller for risk scores with fewer parameters, which were derived only from measured parameters (scores S5 and S7 in Table 6). The overestimation demonstrated by the scores with medical diagnoses can be explained by the fact that all medical diagnoses are in fact already characterized by the measured parameters. The moderate FPR value reflected the fact that the control group did not consist of absolute healthy individuals. All patients without IVH who had high score values (Figure 2 right), had also elevated CRP level (greater than 10 mg/L) indicating an inflammation. In this case, although they did not have IVH, they could not be regarded as healthy patients either.

**Table 6:** Performance of the constructed IVH risk scores on the validation cohort.

Score	AUC		TPR		FPR
	IVH grades		IVH grades		
	I–IV	III–IV	I–IV	III–IV	
S1	0.64	0.69	0.51	0.61	0.42
S2	0.57	0.67	0.77	0.86	0.72
S3	0.58	0.67	0.80	0.86	0.64
S4	0.79	0.85	0.72	0.88	0.64
S5	0.79	0.85	0.72	0.94	0.48
S6	0.79	0.85	0.75	0.88	0.52
**S7**	**0.79**	**0.85**	**0.75**	**0.94**	**0.48**

Key information in the table is highlighted in bold.

## 4 Discussion

IVH is one of the most dangerous pathologies of preterm birth leading to serious lifelong disabilities. The origin and progression of IVH has a multifactorial background. The present study focused on statistical analysis of various medical factors associated with IVH which then were used for construction of a scoring system for prediction of IVH risk. Using a stepwise multivariable logistic regression analysis, several risk scores were constructed for different clinical situations. A particular novelty of the present research was the inclusion of mathematically calculated CBF as an independent predictor variable in the construction of IVH risk scores.

The IVH risk score based only on prenatal and birth parameters, Apgar values and medical diagnoses (S1) has demonstrated only moderate performance, indicating that they are insufficient for effective IVH prediction. A considerable improvement in the prediction of IVH was achieved by including the measured parameters into the logistic regression model. As a result, risk scores constructed using parameters averaged over the first day of life (S2 and S3) and over all days before IVH (S4–S7) demonstrated TPR = 0.9 for severe IVH both in the derivation and validation cohorts.

The best performance in the validation cohort with AUC = 0.85 and TPR = 0.94 for severe IVH, AUC = 0.79 and TPR = 0.75 for all IVH grades, and FPR = 0.48 for cases without IVH was demonstrated by the risk score S7 which included CBF. The advantages of CBF as an independent predictive variable were confirmed by several statistical methods. Thus, the calculated CBF was ranked fourth among statistically significant parameters and had a stronger association with IVH than pCO_2_, pO_2_ and Ht, which were used for its calculation. Furthermore, the superiority of the risk score with CBF as an independent parameter was also demonstrated by comparison with a risk score constructed without CBF but taking into account all clinical parameters used in the mathematical model to calculate CBF. The better performance of risk assessment using CBF was evidenced by a higher AUC value compared with the AUC for risk assessment without CBF (0.94 vs. 0.86), which proves that the mathematical model for calculating CBF provides additional information that improves IVH risk prediction.

The moderate FPR value reflects the fact that the control group did not consist of completely healthy patients. For example, all patients without IVH but with high scores had elevated CRP levels indicating inflammation. Thus, constructed risk scores may reveal additional morbidity risks for the preterm infant. Since the main purpose of the constructed scores is to identify patients at risk, the obtained FPR values are acceptable for this situation. The absence of medical diagnoses in the best score can be explained by the argument that all medical diagnoses are in fact characterized by the measured parameters.

The focus of the present work was on the role of routinely measured clinical parameters and calculated CBF in the development of a risk scores for IVH prediction. The importance of measured parameters in the early detection of IVH risk in low birth weight newborns (<1,500 g) was also demonstrated in Huvanandana et al. (16) by scoring models derived using time series analyses of blood pressure and respiratory data. In our study, the developed IVH risk scales demonstrated in both the derivation and validation cohorts the high performance, which is comparable to data published in the literature. In Chien et al. (12), the standard SNAP II mortality risk score (9) combined with GA and Apgar at 5 min demonstrated an AUC = 0.8 for IVH prediction in a cohort of 4,226 infants with GA < 32 weeks. Heuchan et al. (37) developed a novel prognostic model for IVH prediction based on five predictive variables (GA, antenatal corticosteroids, transfer after birth, Apgar at 1 min < 4, male gender) that demonstrated AUC = 0.76 for severe IVH and AUC = 0.67 for any grade of IVH on a cohort of 5,712 infants with gestation of 24–30 weeks. In Vogtmann et al. (1), in cohort of 1,782 neonates with GA < 32 completed weeks of gestation or BW < 1,500 g, severe IVH could be predicted with an accuracy of 87.7% on the basis of five variables (GA, pathological Doppler result, Apgar at 1 min < 6, perinatal infection, and delivery mode). A scoring system including only three factors (low GA, low Apgar at 5 min, and presence of bleeding diathesis within the 7 days of life) developed by Coskun et al. (14) could predicted IVH in preterm infants with GA between 24 and 34 weeks with AUC = 0.85 for a derivation cohort with 144 preterm infants and AUC = 0.81 for a validation cohort with 89 preterm infants.

In the present study, several risk scores based on routinely measured parameters and calculated CBF were developed. They have demonstrated better performance compared to risk models based only on once-measured parameters (like Apgar) and medical diagnoses. In addition, the developed IVH risk scores provide dynamic information about the patient’s condition, which can vary from hour to hour. This indicates the need for further investigation of possible prognostic variables among clinical parameters. Also, medical treatment may be considered in the further development of the risk models.

At present, many researchers are investigating neonatal cerebral hemodynamics using modern techniques such as transcranial Doppler (TCD) ultrasound and NIRS monitoring to predict periventricular-intraventricular hemorrhage in preterm infants (38, 39). Although a strong association between IVH and low superior vena cava flow (SVCF) and high Anterior cerebral artery resistive index (ACA-RI) has been revealed (38) the absolute values of global CBF cannot be recalculated from measurements because it is difficult to determine the diameter of intracranial vessels (38, 40). In this case, the mathematical calculation of CBF provides an opportunity to obtain additional information that may improve the prediction of IVH in preterm infants. The developed mathematical model for CBF calculation was previously validated against NIRS, Doppler and Xenon-133 clearance measurements and demonstrated good agreement with published experimental results (19). Furthermore, the current study showed that the inclusion of calculated CBF in IVH risk models resulted in improved performance of prognostic scores. Therefore, in the future, when CBF measurement becomes a routine procedure in neonatal healthcare, the developed risk scores can be used with the measured data.

Nowadays, numerous data and parameters are collected and centrally managed on admission to the NICU. This already allows for automated computerized data analysis (41). Existing health monitoring methods can be further advanced with new strategies, such as the mathematical model for calculating CBF and the IVH risk scores presented in this paper. The good performance of the developed scores allows their use in clinical practice as a complementary tool to other clinical scores and measurement methods to identify preterm infants at high risk of severe IVH, which may lead to a more effective therapeutic approach for these children.

## Data Availability

The data analyzed in this study is subject to the following licenses/restrictions: raw data supporting the conclusions of this manuscript will be made available to any qualified researcher upon request to the corresponding author. Requests to access these datasets should be directed to renee.lampe@tum.de.
